# Supplementary material for: Chromothripsis during telomere crisis is independent of NHEJ, and consistent with a replicative origin
Source: Genome Res. 2019 May;29(5):737–49. doi: 10.1101/gr.240705.118 (PMC6499312; doi:10.1101/gr.240705.118)
Supplement: Supplemental Material [file supp_gr.240705.118_Supplemental_file_1.zip › contigs/annotated_contigs/DB57/contig.2.DB57_length_366_mean_cov_3.29781420765.docx]

**DB57_length_366_mean_cov_3.29781420765**

```
AATGATGGATTCCTGCATATAAATGTATAGTTTATTAATGTAATGGAGTCACATTGCCGTGGAAGAATTCTTCCTAATTTTATGCCAGG
 >chr9:107252886-107253122 - E=1e-127
TTAACTATGCTTCTGCAGAGAACACTGCATCTTTCAGATGCTCTGCCAATGGATTTAAGTGTGAGACCGGCTAGTATAAGAGAATGCAA

CCACATATCTGGCCGGAGAACTGCCAGTTAAAATATATATTACTAAAAATAGCCCT|CT|TTGTCTAAATTTCACCATTATCCTCAGCA
                                                         >chr9:107235209-107235341 - E=
ATCATTAGACTTGTGGAAAGTCACAGTTTTGAAGAAAAGTTTTGGATTTGTTTCCTGAGAAAATATGAATGCCTTAATGAATTTATTGC
5e-68
TTAGTTCCGTCT
```
